# Supplementary material for: Selectively tunable optical Stark effect of anisotropic excitons in atomically thin ReS2
Source: Nat Commun. 2016 Nov 18;7:13569. doi: 10.1038/ncomms13569 (PMC5120211; doi:10.1038/ncomms13569)
Supplement: Supplementary Information — Supplementary Figures 1-7, Supplementary Notes 1-3 and Supplementary References [file ncomms13569-s1.pdf]

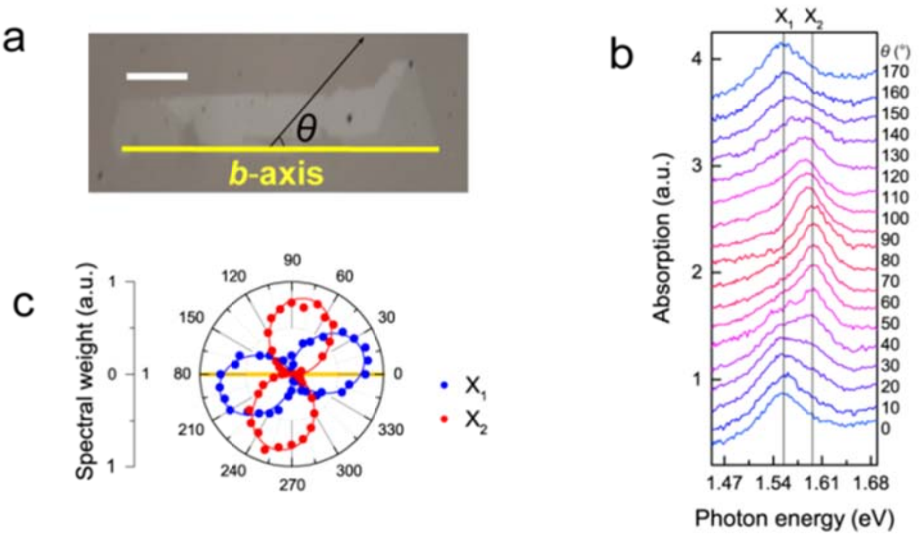

Supplementary Figure 1 | Light-polarization-dependent absorption of bilayer ReS_2. (**a**) optical image of bilayer ReS_2. Thick yellow line indicates the crystal *b*-axis. Scale bar, 25 μm**.** (**b**) Polarization-dependent absorption spectra of bilayer ReS_2. (**c**) Corresponding spectral weights of Lorentzian contributions of X_1 (blue dots) and X_2 (red dots). Solid fit lines are proportional to $\cos^2(\theta-\theta_{max})$ with offset, where θ_{max} values are polarization angles of the excitons (16° for X_1 and 78° for X_2).

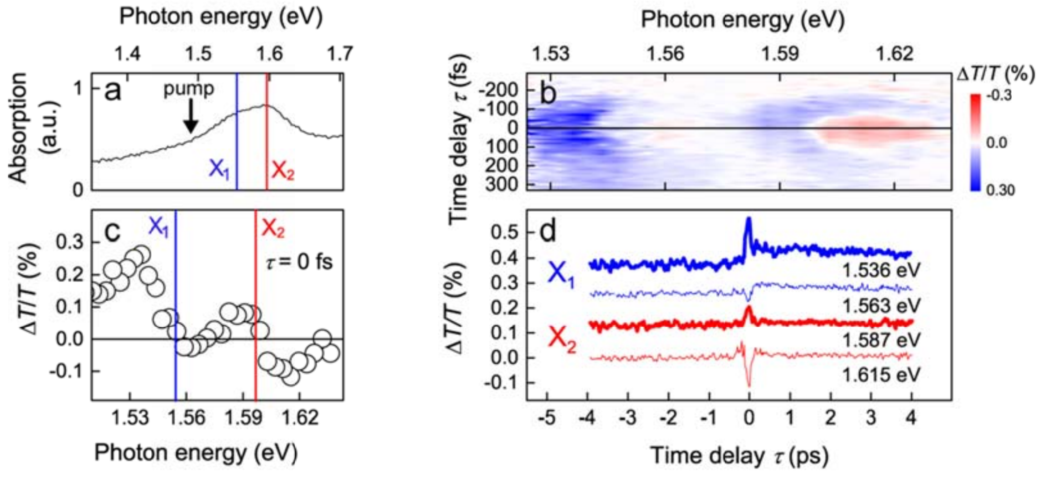

Supplementary Figure 2 | Observation of the optical Stark effect in bilayer ReS_2 at room temperature. (**a)** Absorption spectrum of bilayer ReS_2 with circularly polarized light. The black arrow indicates the pump photon energy of 1.49 eV for the following pump-probe measurements. (**b)** Transient DT spectra with linear pump polarization of $\theta = 40°$. (**c)** Corresponding DT spectrum at $\tau = 0$ (circles). Blue (red) vertical lines in (**a**) and (**c**) show the energy of the X_1 (X_2) exciton state without pump. (**d**) DT time-traces near X_1 (blue) and X_2 (red) exciton energies. Corresponding probe photon energies are shown on the right.

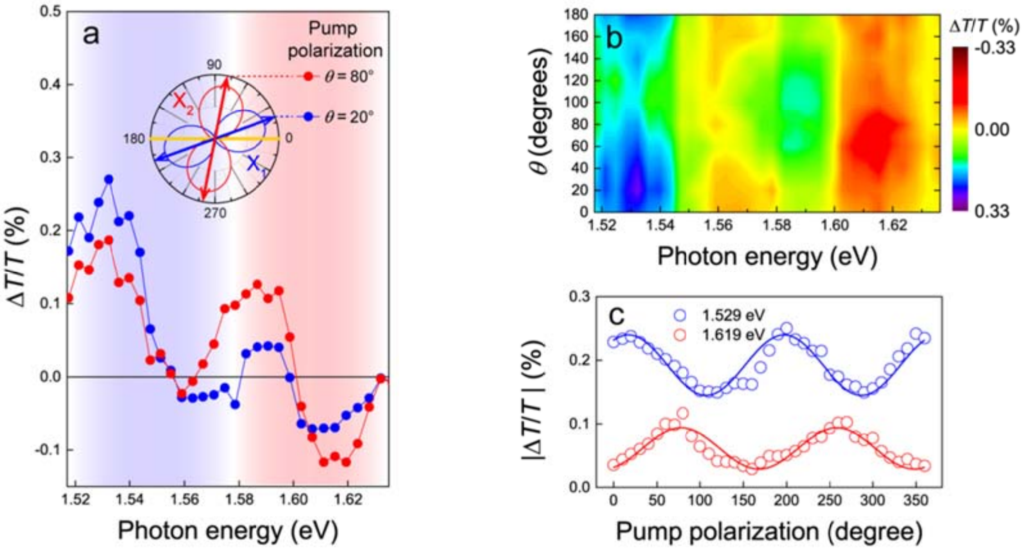

Supplementary Figure 3 | Pump-polarization-dependent optical Stark effect of excitons in bilayer ReS_2 at room temperature. (**a**) DT spectra at τ = 0 fs of linearly polarized optical excitations with two important polarization angles of θ = 20° (blue) and 80° (red), at which the pump light is closely aligned to X_1 and X_2, respectively. The probe is circularly polarized. The blue- (red-) shaded area represents the spectral region where the DT signal is dominated by the shift of the X_1 (X_2) state. Inset: light-polarization-dependent absorption spectral weights of X_1 (blue) and X_2 (red) (arbitrary units). The yellow line indicates the *b*-axis direction. Double-sided arrows show the angles of the pump-polarizations of the DT spectra in the main panel. (**b**) Pump-polarization-dependent DT spectra at τ = 0. (**c**) Corresponding absolute DT values at 1.529 eV (blue circles) and at 1.619 eV (red circles). Solid lines are fits.

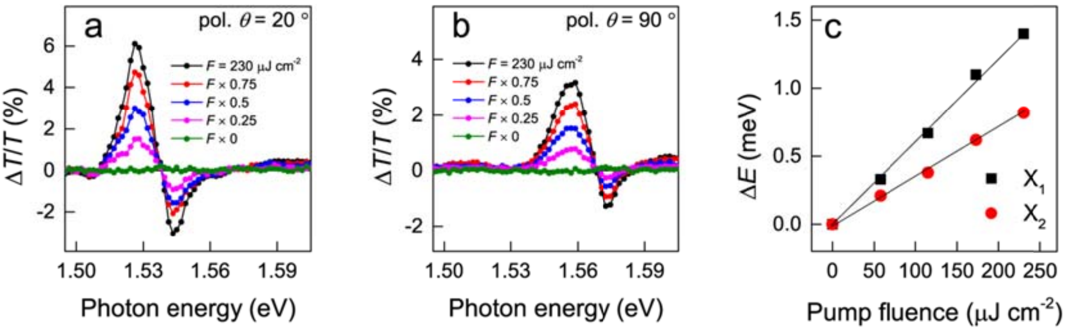

Supplementary Figure 4 | Pump-fluence dependence of the exciton shifts of few-layer ReS_2 at 78 K. (**a**,**b**) Pump-fluence-dependent DT spectra at τ = 0 fs with co-linear pump-probe polarization configurations at θ = 20° (**a**) and θ = 90° (**b**). Pump photon energy is fixed at 1.44 eV. (**c**,**d**) Corresponding pump-induced shift of X_1 (**c**) and X_2 (**d**). Solid lines are guides to the eye.

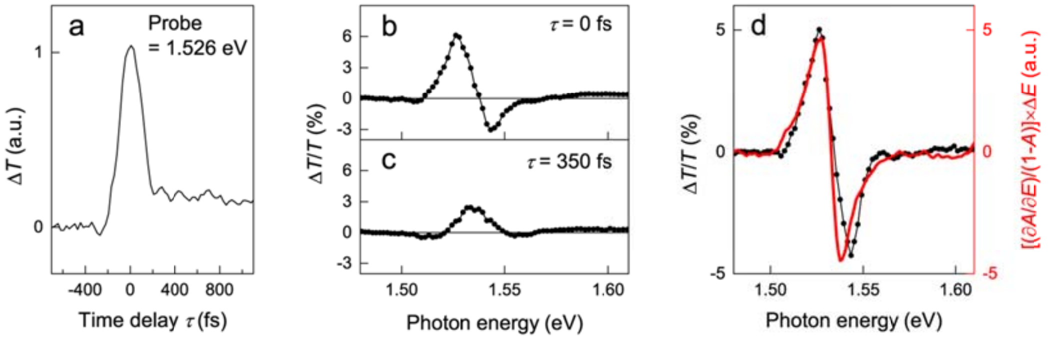

Supplementary Figure 5 | Estimation of ΔE_1 of few-layer ReS_2 at 78 K. (**a**) DT trace at the fixed probe photon energy of 1.526 eV. Polarizations of pump and probe are both $\theta = 20°$. (**b,c**) Corresponding DT spectrum at $\tau = 0$ fs (**b**) and 350 fs (**c**). Note that (**b**) is identical to the middle panel of Fig. 3a in the main text. (**d**) Black dots are background-subtracted DT spectrum (left axis) and red solid line is $\left(\partial A_{\text{no pump}} / \partial E\right) / \left(1 - A_{\text{no pump}}\right)$ scaled by ΔE (right axis).

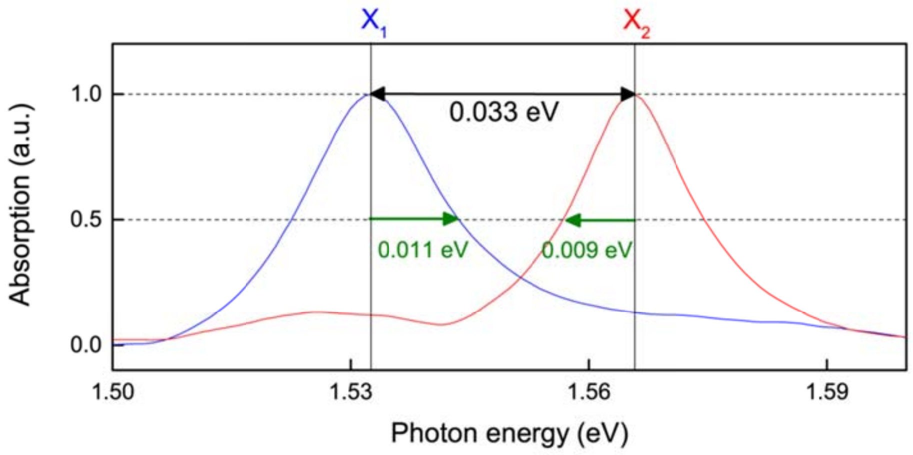

Supplementary Figure 6 | Spectral distance between X_1 and X_2. Blue and red lines are background subtracted absorption spectra of few-layer ReS_2 at 78 K with light polarization of $\theta = 20°$ (blue) and $\theta = 90°$ (red). Original spectra are shown in the top panels of Fig. 3a and 3b of the main text. Black arrow shows spectra distance between X_1 and X_2. Green arrows indicate their half linewidths. We can see that the spectral distance between X_1 and X_2 excitons (0.033 eV, black arrow) is about 1.65 times larger than the sum of their half linewidths (0.011 eV + 0.009 eV, green arrows), meaning that the two exciton levels are well separated. This fact further justifies that our findings in Fig. 3 of the main text possess energy-selectivity for the excitonic optical Stark effect.

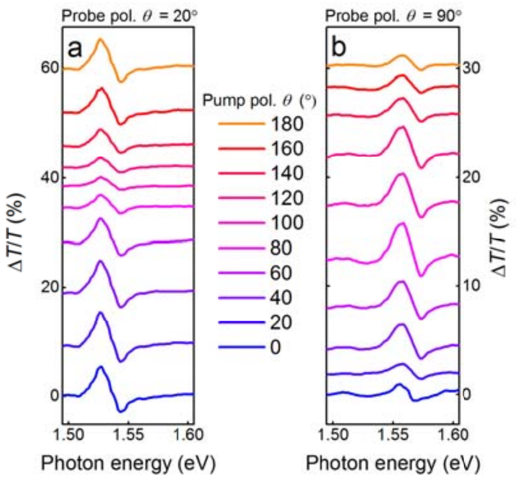

Supplementary Figure 7 | Pump-polarization-dependent DT spectra. (**a,b**) Pump-fluence-dependent DT spectra at $\tau = 0$ fs with probe polarization of $\theta = 20°$ (**a**) and $\theta = 90°$ (**b**). Pump photon energy is fixed at 1.44 eV. Offset for clarity.

Supplementary Note 1. Tunable optical Stark effect in bilayer ReS_2 at room temperature.

In this section, we present optical Stark effect of excitons in bilayer ReS_2 at room temperature (see Supplementary Figure 1a for the optical image of the bilayer sample). Similar to the results in the main text, excitons in bilayer ReS_2 also show pump-polarization-dependent optical Stark shifts as follows.

In this experiment, unlike the main text, while we used polarization-controlled linear pump pulses to excite the sample, we used circularly polarized pulses (possessing the whole electric-field directions) to probe DT. The reason for this was to focus solely on the influence of the pump polarization on the system's optical response, and to experimentally exclude the contribution of probe light on the measured polarization-dependent signals. However, unlike the discussion in the main text (where linearly polarized probe beams are used to selectively measure the shift of one exciton state, see Fig. 3 in the main text), such measurements

requires many parameters for estimating the shifts of excitons because both X_1 and X_2 have effects on DT spectrum[1]. For this reason, we analyze the data in this section without estimating ΔE.

We first measured the light-polarization dependent equilibrium absorption spectra, as shown in Supplementary Figure 1b. Similar to the few-layer sample (Fig. 1d in the main text), the absorption peaks (X_1 and X_2) arise from the two exciton states and show strong polarization dependence[2]. Corresponding θ -dependent Lorentzian spectral weights are shown in Supplementary Figure 1c, where the excitons are polarized at different angles (θ ~ 16° and ~ 78° for X_1 and X_2, respectively).

We now explore the detailed DT response of bilayer ReS_2 with fixed pump polarization (θ = 40°) to confirm the excitonic optical Stark effect. Supplementary Figure 2a shows the equilibrium absorption spectrum with circularly polarized light. Two absorption peaks due to X_1 and X_2 are observed near 1.55 eV and 1.60 eV, respectively (see vertical lines)[2]. To measure the optical Stark shifts of these two states, we excited the sample with pump photon energy detuned to 60 meV below X_1 transition (see black arrow in Supplementary Figure 2a) and monitored the time-resolved DT spectra, as shown in Supplementary Figure 2b. Here, the line-cut DT spectrum at $\tau = 0$ fs (circles in Supplementary Figure 2c) clearly shows positive-to-negative sign changes near X_1 and X_2, indicating optical Stark shifts of both excitons. This is corroborated by the DT time-traces (Supplementary Figure 2d).

Next, we investigate how the optical Stark effects of the excitons change depending on light polarization. For this purpose, we measured the DT spectra at τ = 0 while varying the polarization (θ) of the pump with photon energy detuned to 60 meV below the X_1 level and fluence fixed at $0.42\,\mathrm{mJ\,cm^{-2}}$. First, we compare the two representative DT responses at θ =

20° and θ = 80°, at which the pump polarizations are closely aligned along X_1 (~ 16°) and X_2 (~ 78°), respectively. The main panel of Supplementary Figure 3a clearly shows that when the spectral region is dominated by the optical Stark shift of X_1 resonance (blue-shaded area), the amplitude of the DT signals at θ = 20° (blue dots) is larger than that at θ = 80° (red dots). On the contrary, in the X_2-dominated spectral region (red-shaded area), the DT amplitude at θ = 80° shows a larger signal compared to that at θ = 20°. This result implies that the optical Stark effect is strong when the orientation of pump light is parallel with the polarization of each exciton, agreeing well with results in the main text.

We also measured the pump-polarization-dependent DT spectra by tuning the angles of pump polarization continuously at τ = 0 fs (Supplementary Figure 3b). In Supplementary Figure 3c, we plot corresponding absolute DT values at 1.529 eV (1.619 eV), at which the response is dominated by X_1 (X_2). As indicated by the solid lines in Supplementary Figure 3c, their gradual changes as a function of θ can be well fit with a simple function $\Delta E(\theta) = a + b\cos^2(\theta - \theta_{\max})$, setting $\theta_{\max}$ to the individual excitons' original polarizations (i.e., $\theta_{\max}$ = 16° for X_1 and $\theta_{\max}$ = 78° for X_2). These results are well in accordance with the behavior of the few-layer sample in the main text. A notable fact is that DT for both exciton states is not negligible even when the pump polarization is orthogonal to $\theta_{\max}$, which is a feature not seen in the absorption spectral weight (Supplementary Figure 1c). This may be because the two excitonic transitions share either the same conduction band or valence band which forms the exciton states[1,3,4].

Supplementary Note 2. Pump-fluence-dependent shifts of exciton levels.

Supplementary Figure 4a (4b) shows the pump-fluence-dependent DT spectra at τ = 0 fs

under the same conditions described in the caption of the middle panel of Fig. 3a (3b) in the main text. We see that corresponding magnitudes of the shift in X_1 (X_2) resonance shows characteristic linear-dependence on the pump fluence, as shown in Supplementary Figure 4c. Considering the relation of $\Delta E \propto \varepsilon^2 \propto F$ (where ε is electric field intensity; F is pump fluence, see main text), these results further confirm that the measured DT arises from the optical Stark effect of excitons[5].

Supplementary Note 3. Estimation of the optical Stark effect from the DT response

In this section, we discuss the estimation procedure of the optical Stark shift (ΔE). Generally, DT is given by[6].

$$\frac{\Delta T}{T}(E) \approx \frac{A_{\text{no pump}}(E) - A_{\text{with pump}}(E)}{1 - A_{\text{no pump}}(E)}, \tag{1}$$

where $A_{\text{no pump}}(E)$ ($A_{\text{with pump}}(E)$) is absorption without (with) pump excitation. When blue-shift of an exciton dominates the DT response, $A_{\text{with pump}}(E)$ can be approximated by $A_{\text{no pump}}(E - \Delta E)$, which yields[7]

$$\frac{\Delta T}{T}(E) \approx \frac{\left[A_{\text{no pump}}(E - \Delta E) - A_{\text{no pump}}(E)\right]}{-\Delta E} \frac{\Delta E}{1 - A_{\text{no pump}}(E)} \approx \frac{\partial A_{\text{no pump}}}{\partial E} \frac{\Delta E}{1 - A_{\text{no pump}}}. \tag{2}$$

This result tells us that the DT arising from the optical Stark effect resembles the derivative of absorption, as discussed in the main text (see Fig. 2b in the main text). In addition, we can see that the magnitude of the shift (ΔE) can be estimated by comparing the measured DT lineshape with the $(\partial A_{\text{no pump}} / \partial E)/(1 - A_{\text{no pump}})$ spectrum scaled by ΔE (where ΔE is only a

free parameter)[8,9].

As an example, let us trace the procedure for estimating the blue-shift of X_1 (ΔE_1) from the measured DT spectrum in the middle panel of Fig. 3a (note that Supplementary Figure 5b shows same graph). Although the optical Stark effect dominates the DT response, its somewhat asymmetric (Supplementary Figure 5b) with a small deviation from the derivative of absorption, considering that the derivative of a single exciton resonance is generally symmetric. This is because the DT response at $\tau = 0$ fs is affected by pump-excited real carriers, which are generated by two-photon- or phonon-mediated-absorption of pump photons[5,10]. Such a mixed response is corroborated by the DT trace in Supplementary Figure 5a, where a spike-like peak at $\tau = 0$ fs due to the optical Stark effect is followed by long-lasting signals originating from pump-generated carriers. Indeed, the transient DT spectrum directly after the optical Stark shift (at $\tau = 350$ fs, see Supplementary Figure 5c) shows a typical bleaching-like shape of X_1 transition[11]. In order to distinguish the bleaching effect from the Stark shift, we subtract the DT at $\tau = 350$ fs (Supplementary Figure 5c) from the DT at $\tau = 0$ fs (Supplementary Figure 5b)[7], producing an almost symmetric shape, as indicated by black dots in Supplementary Figure 5d. This background-subtracted DT spectrum now closely follows the lineshape of $(\partial A_{\text{no pump}} / \partial E)/(1 - A_{\text{no pump}})$ (red line in Supplementary Figure 5d), which enables us to estimate ΔE by comparing two graphs in Supplementary Figure 5d.

Supplementary References

1. Choi, C. K. *et al.* Polarization dependence of the excitonic optical stark effect in GaN. *Phys* **65,** 155206 (2002).

2. Aslan, O. B., Chenet, D. a., van der Zande, A. M., Hone, J. C. & Heinz, T. F. Linearly polarized excitons in single- and few-layer ReS_2 crystals. *ACS Photonics* **3,** 96–101 (2016).

3. Gupta, J. a, Knobel, R., Samarth, N. & Awschalom, D. D. Ultrafast manipulation of electron spin coherence. *Science.* **292,** 2458–2461 (2001).

4. Joffre, M., Hulin, D., Migus, A. & Combescot, M. Laser-induced exciton splitting. *Phys. Rev. Lett.* **62,** 74 (1988).

5. Von Lehmen, A., Chemla, D. S., Zucker, J. E. & Heritage, J. P. Optical Stark effect on excitons in GaAs quantum wells. *Opt. Lett.* **11,** 609–611 (1986).

6. Schmidt, R. *et al.* Ultrafast Coulomb intervalley coupling in atomically thin WS_2. *Nano Lett.* **16,** 2945 (2016).

7. Sie, E. J. *et al.* Valley-selective optical Stark effect in monolayer WS_2. *Nat. Mater.* **14,** 290 (2014).

8. Song, D. *et al.* Measurement of the optical Stark effect in semiconducting carbon nanotubes. *Appl. Phys. A* **96,** 283–287 (2009).

9. Kim, J. *et al.* Ultrafast generation of pseudo-magnetic field for valley excitons in WSe2 monolayers. *Science* **346,** 1205 (2014).

10. Knox, W. H., Chemla, D. S., Miller, D. a B., Stark, J. B. & Schmitt-Rink, S. Femtosecond ac Stark effect in semiconductor quantum wells: Extreme low- and high-intensity limits. *Phys. Rev. Lett.* **62,** 1189–1192 (1989).

11. Sim, S. *et al.* Exciton dynamics in atomically thin MoS_2: Interexcitonic interaction and broadening kinetics. *Phys. Rev. B* **88,** (2013).
